# The Role of Endoplasmic Reticulum Stress in the Development of Periodontitis—From Experimental Cell and Animal Models to Humans

**DOI:** 10.3390/ijms26199620

**Published:** 2025-10-02

**Authors:** Sebastian Gawlak-Socka, Paulina Sokołowska, Gabriela Henrykowska, Edward Kowalczyk, Sebastian Kłosek, Anna Wiktorowska-Owczarek

**Affiliations:** 1 Department of Pharmacology and Toxicology, Medical University of Lodz, Żeligowskiego 7/9, 90-752 Lodz, Poland; sebastian.gawlak-socka@student.umed.lodz.pl (S.G.-S.); paulina.sokolowska@umed.lodz.pl (P.S.); edward.kowalczyk@umed.lodz.pl (E.K.); 2 Department of Epidemiology and Public Health, Medical University of Lodz, Żeligowskiego 7/9, 90-752 Lodz, Poland; gabriela.henrykowska@umed.lodz.pl; 3 Department of Oral Pathology, Medical University of Lodz, Pomorska 251, 92-213 Lodz, Poland; sebastian.klosek@umed.lodz.pl; 4 Department of Periodontology and Oral Mucosal Diseases, Medical University of Lodz, Pomorska 251, 92-213 Lodz, Poland

**Keywords:** periodontitis, ER stress, UPR pathway

## Abstract

Periodontal disease is a prevalent inflammatory disorder that can lead to severe oral complications. Recent studies increasingly underline the role of endoplasmic reticulum (ER) stress in its pathogenesis. Experimental models using inflammatory agents such as lipopolysaccharide (LPS), tumor necrosis factor-alpha (TNF-α), and ligature-induced periodontitis in rodents, as well as chemical hypoxia, have consistently demonstrated the activation of unfolded protein response (UPR) pathways in periodontal cells. Key ER stress markers, including CHOP, GRP78, PERK, and ATF6, were upregulated in periodontal ligament cells, stem cells, and gingival epithelial cells under these conditions. While ER stress in periodontitis is primarily associated with detrimental outcomes such as apoptosis and inflammation, it may also have a physiological role in bone remodeling via the PERK-eIF2α-ATF4 axis. Importantly, several ER stress-modulating agents—such as oridonin, melatonin, and exosomes derived from M2 macrophages—have shown therapeutic potential by reducing stress marker expression and limiting periodontal damage. These findings suggest that targeting ER stress may offer a novel therapeutic strategy. Future human studies are essential to determine whether a combined approach targeting inflammation and ER stress could more effectively halt or reverse periodontal tissue destruction, while also assessing the long-term safety of ER stress modulation.

## 1. Introduction

Periodontitis is considered a social oral disease, primarily due to its high prevalence within the human population. According to studies conducted between 2009 and 2014 in the United States, 42% of adults were affected by this condition, with a lower percentage observed for its severe form [1]. Research conducted in 2017 demonstrated that the age-standardized prevalence of severe periodontitis was 9.8 cases per 100 individuals [2]. These studies also highlighted a global trend toward lower prevalence rates of severe periodontitis, particularly in middle-income populations. In contrast, in the wealthiest countries, an increase in the prevalence rate has been observed over the past three decades [2].

Periodontitis is a chronic inflammatory disease, characterized by the progressive loss of alveolar bone and connective tissue attachment, leading to irreversible destruction of the supporting tissues of the teeth, resulting in tooth mobility and eventual loss [3]. It is associated with other medical conditions and may predispose individuals to the development of oral cancer. Tezal et al. [4] demonstrated an increased incidence of oral cancers in patients with clinical attachment loss (CAL) greater than 1.5 mm. Al-Hebshi et al. [5] showed that periodontal pathogens, such as *Porphyromonas gingivalis* and *Fusobacterium nucleatum*, are frequently isolated from oral cancer tissues. Additionally, periodontitis has been linked to an increased risk of Alzheimer’s disease and mild cognitive impairments [6] and contributes to the development of various systemic diseases, including diabetes and cardiovascular diseases [7,8]. Although COVID-19 is solely caused by the SARS-CoV-2 virus and periodontitis does not contribute to its development, emerging evidence suggests that the presence of periodontitis may exacerbate the severity of COVID-19 symptoms [9]. This association highlights the potential impact of oral health on the course of systemic infections and supports the importance of periodontal care, especially in patients with comorbidities.

Due to the high prevalence of periodontitis, the complications associated with this periodontal disease, and its potential to trigger or exacerbate other systemic diseases, it is crucial to gain a detailed understanding of the pathomechanisms underlying this condition. The primary cause of periodontitis is the accumulation of dental plaque and the presence of pathogenic bacteria, particularly those from the red complex, on subgingival surfaces. These microorganisms trigger an inflammatory response, which, although not the cause of the disease itself, plays a central role in the subsequent tissue destruction observed at both the histological and cellular levels [3]. Therefore, understanding additional mechanisms contributing to the development of periodontitis, such as endoplasmic reticulum stress (ER stress), may be significant for identifying new drug targets and enabling faster, more effective treatments. This article explores how inflammation, a central factor in periodontitis development, may trigger ER stress, which in turn influences disease progression. Specifically, the authors elucidate the role of ER stress in the development of periodontitis by demonstrating activation of the unfolded protein response (UPR), as evidenced by the elevated expression of key markers such as PERK, ATF6, CHOP and GRP78.

## 2. Periodontitis Causes

Periodontal inflammation is a multifactorial inflammatory disease, in which dental plaque, and particularly the bacteria accumulated within it, play a central role. It is important to note that not all bacteria present in dental plaque, e.g., *Rothia dentocariosa* and *Streptococcus gordonii*, are associated with periodontitis [10,11]. The development of periodontitis depends not only on the presence of bacteria but also on the order of their colonization and their virulence mechanisms.

During plaque maturation, early colonizers such as *Streptococcus oralis* and *Streptococcus gordonii* initiate biofilm formation by adhering to the pellicle. These bacteria create conditions that favor the adhesion of secondary colonizers, including *Fusobacterium nucelatum* and *Capnocytophaga* spp., which serve as bridging organisms between early and late colonizers. With the reduction of oxygen tension in the maturing plaque, strictly anaerobic pathogens gain an advantage [12,13].

**Figure 1:**
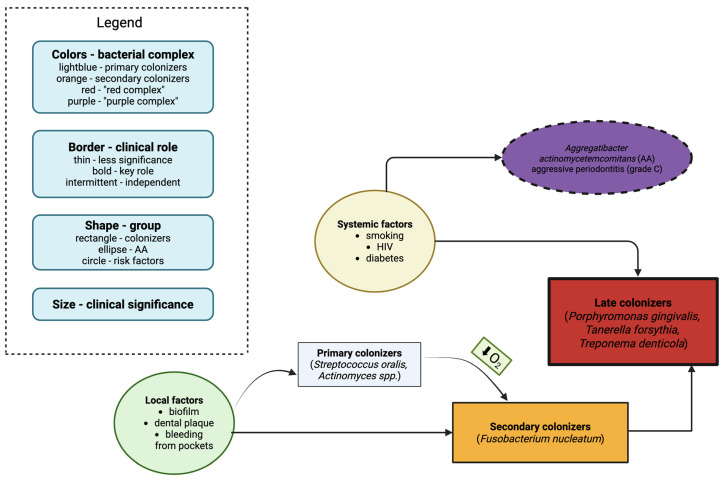
Bacterial complexes in periodontitis. The colors represent distinct pathogen groups involved in the development of periodontitis. Pathogens in the purple complex possess multiple potent virulence factors that induce cell death and either trigger or evade inflammatory responses. The red complex includes pathogens with a strong inflammatory capacity. The orange complex comprises pathogens crucial for the initiation of periodontal disease. The pathogens listed in the light blue rectangle are primary colonizers of dental plaque. Oxygen reduction leads to the development of anaerobic bacteria, including secondary colonizers. Created in BioRender Gawlak-Socka, S. (2025) https://BioRender.com/4qv9w7c [19].

Late colonizers include bacteria of the “red complex”—*Porphyromonas gingivalis*, *Tannerella forsythia*, and *Treponema denticola*—considered the key pathogens associated with destructive periodontal disease due to their ability to evade host immune defenses and induce tissue destruction [13]. Another important species is *Aggregatibacter actinomycetemcomitans*, which belongs to the “purple complex”, particularly relevant in aggressive periodontitis (grade C) (Figure 1). Unlike other pathogens, *A. actinomycetemcomitans* does not fully rely on the standard colonization sequence and can adhere directly to the tooth surface or epithelium via adhesins, fimbriae, and outer membrane vesicles [14,15,16]. This bacterium produces leukotoxin, which impairs neutrophil function and is strongly associated with rapid periodontal breakdown in young individuals [16]. These bacteria are most commonly associated with this disease, mainly because they effectively colonize the host organism, have developed mechanisms to evade host defense responses, and cause damage to host cells [14]. In particular, *P. gingivalis*, for which the anaerobic conditions present in the periodontium provide an ideal environment for colonization, can adhere even in the early stages of dental plaque development associated with periodontitis [15]. *T. forsythia* forms a synergistic biofilm with other bacteria responsible for periodontitis and can effectively evade the host immune response, making it one of the key factors in the development of this disease [17,18].

Not only are anaerobic bacteria associated with the onset of periodontitis, but certain facultatively aerobic bacteria, such as *Eikenella corrodens*, *Capnocytophaga* spp., *Streptococcus oralis*, *Staphylococcus aureus*, *Pseudomonas aeruginosa*, and *Enterococcus faecalis*, also play a role [20,21,22].

Although bacterial factors represent the main etiological agents, the disease is also shaped by local and systemic factors. Local factors include unfavorable dental anatomy (tooth crowding, rotations, malpositions) that promote plaque retention and hinder effective oral hygiene [23,24,25,26,27]. The systemic factors presented in Table 1 also play a crucial role.

Additionally, genetic predisposition is an important modifying factor. Mutations and polymorphisms in TLR4, TLR9, cytokine genes (IL-1, IL-6, TNF-α) and matrix metalloproteinases (MMP-3, MMP-9) have been linked with dysregulated inflammatory responses and enhanced tissue destruction [24,38].

Finally, social and nutritional factors, such as poor socioeconomic status or deficiencies in micronutrients (e.g., vitamin C, calcium), can impair host defenses and indirectly increase disease risk [39,40].

In summary, while bacterial colonization and plaque maturation are fundamental to disease initiation, the interplay with local, systemic, genetic and social factors determines disease severity and progression.

### Periodontitis Pathomechanism

**Figure 2:**
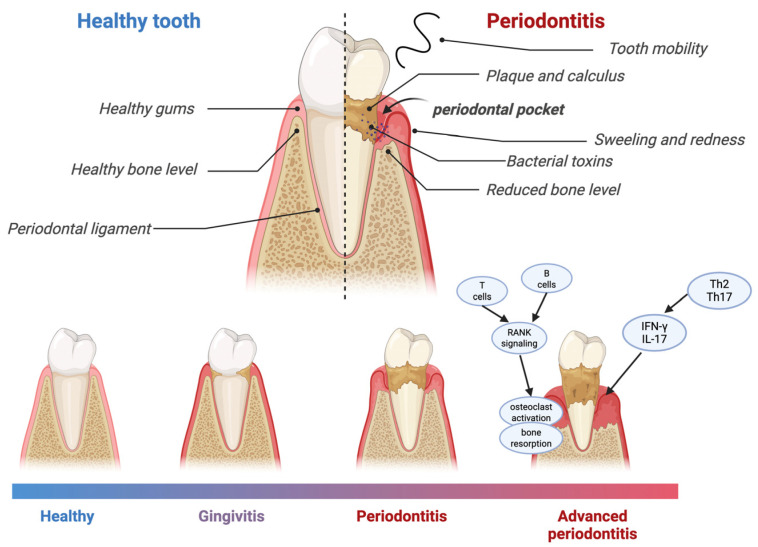
An anatomical illustration of healthy periodontium and periodontitis, resulting from the interplay of local and systemic factors. As the disease progresses, the inflammatory response intensifies, leading to deepening of the periodontal pocket, reduction in the periodontal ligament, and loss of alveolar bone. This condition is mediated by a range of immune responses, including activated Th2 and Th17 lymphocytes, which produce pro-inflammatory cytokines that contribute to tissue damage. Additionally, T and B cells produce RANKL, which activates osteoclasts, thereby promoting alveolar bone resorption. Created in BioRender Gawlak-Socka, S. (2025) https://BioRender.com/gcdnmcc [43].

Pathogenic microorganisms and their products act on gingiva for a long time and cause immune response, which first leads to gingival inflammation. When inflammation spreads to the deep periodontal tissue, causing the dissolution and destruction of collagen fibers in the gingiva and periodontal ligament and absorption of alveolar bone, this results in periodontitis, as illustrated in Figure 2. The main symptoms of periodontitis, which result from ongoing inflammation, are the formation of periodontal pockets, the loss of alveolar bone, and the loosening and loss of teeth [41,42].

Pathogenic microorganisms, by interacting with the gingival tissue over an extended period, cause itching and redness. When the inflammation persists and spreads deeper, it leads to the loss of connective tissue attachment, which is a clinical indicator of periodontitis, as well as to the loss of alveolar bone, resulting in the formation of a periodontal pocket, marking the onset of periodontitis. All these effects of periodontitis can lead to tooth mobility, which, in turn, may result in tooth loss.

The pathomechanism of periodontitis is complex and involves a multitude of inflammatory and immunological factors. The primary etiological agents responsible for this condition are bacteria. As described in the Causes Section, bacteria colonize the dental plaque biofilm sequentially, creating a dysbiotic environment. Key pathogens, including members of the red complex and *A. actinomycetemcomitans*, produce virulence factors that trigger a chronic immune-inflammatory response [14,15,16,17,18]. Bacterial components, such as lipopolysaccharides and leukotoxin, activate the complement system and recruit immune cells, increasing vascular permeability [42,43].

Neutrophils, as the first line of defense, migrate into the gingival crevice, releasing reactive oxygen species and antimicrobial peptides (defensins, LL-37, calprotectin), which control pathogens but also damage host tissues [44,45]. Dendritic cells present antigens to T-helper cells, inducing secretion of pro-inflammatory cytokines (IL-1β, IL-6, TNF-α, IL-17, IL-23) that amplify inflammation and stimulate osteoclast activation [46,47]. B cells and plasma cells infiltrate the tissue, sustaining the inflammatory milieu [42,44].

Inflammatory cytokines induce RANK-L expression on osteoblasts and T cells, which binds to RANK on osteoclast precursors, promoting differentiation into mature osteoclasts and leading to alveolar bone resorption [45,48]. Anti-inflammatory cytokines (IL-10, IL-12, IFN-γ) attempt to regulate this response, but dysregulation results in persistent inflammation and progressive tissue destruction [46,47]. Increased gingival crevicular fluid, swelling and formation of cellular infiltrate consisting mainly of B cells, plasma cells and T-helper subsets (Th2, Th17) contribute to the clinical signs of periodontitis, including periodontal pocket formation, attachment loss and eventual tooth mobility and tooth loos [42,44].

Thus, the pathomechanism of periodontitis reflects a dysregulated host response to a pathogenic biofilm, in which protective immune mechanisms paradoxically drive tissue destruction and disease progression [42,44,45,48,49].

In the immuno-inflammatory response associated with periodontitis, diverse populations of immune cells are involved, with their interactions regulated through cytokine secretion under the control of immunoregulatory mechanisms [50]. Regulation occurs at both the cellular level, via nonspecific responses such as bacterial antigens, antigen-presenting cells, and the hypothalamic–pituitary–adrenal axis, and at the molecular level, through fluctuations in signaling protein concentrations [51].

## 3. Endoplasmic Reticulum Stress (ER Stress)

**Figure 3:**
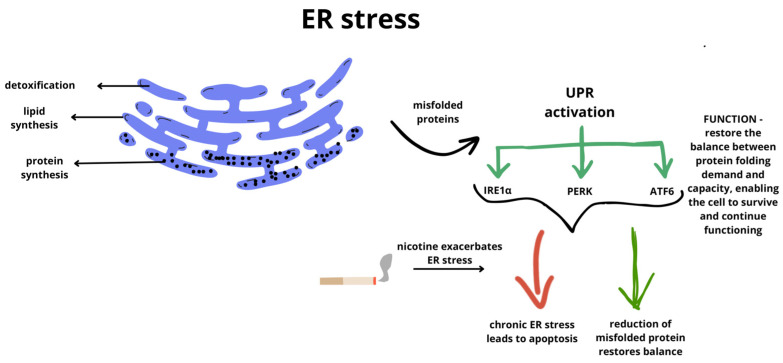
Endoplasmic reticulum stress, leading to the activation of three UPR factors. The sarcoplasmic reticulum is an organelle that can be divided into rough and smooth regions. Under physiological conditions, the smooth endoplasmic reticulum (ER) is responsible for detoxification and lipid synthesis, while the rough ER is involved in protein synthesis. When the environmental conditions of the cell deteriorate, such as exposure to excessive heat or lack of oxygen, it leads to ER stress, resulting in the misfolding of proteins. This triggers the activation of the unfolded protein response (UPR), which, through three signaling pathways—IRE1, PERK, and ATF6—aims to either initiate apoptosis due to chronic ER stress or reduce misfolded proteins to restore balance, depending on the prevailing conditions. Factors such as nicotine present in cigarettes can influence the pathway taken by the UPR.

The ER is an organelle composed of a system of channels isolated from the cytoplasm by biological membranes. It forms an irregular network of vehicles, cisternae, and tubules. This organelle can be divided into two types based on structural differences: rough ER, associated with numerous ribosomes, and smooth ER, which lacks ribosomes (Figure 3). The ER plays multiple roles in the human cells, including protein and lipid synthesis, detoxification of toxins and drugs, intracellular transport, and participation in carbohydrate metabolism [52,53].

Various physiological and pathological conditions can lead to excessive protein synthesis, placing stress on the ER. These include rapid cell proliferation, chronic inflammation, bacterial infection, oxidative stress, and increased metabolic demand. Such conditions elevate the load of nascent proteins within the ER, increasing the likelihood or misfolding and overwhelming the protein quality control mechanisms [52,53,54]. Excessive protein synthesis in the ER can proceed incorrectly, resulting in misfolded or unfolded proteins. Additionally, when there is an accumulation of misfolded or improperly folded proteins, a condition known as ER stress occurs. This condition can be partially alleviated through the activation of the UPR pathway, which stimulates the degradation of misfolded proteins, halts protein translation, and induces the synthesis of molecular chaperones to maintain homeostasis in the human body [54].

The UPR is initiated by three ER transmembrane proteins: Inositol-Requiring Enzyme 1 (IRE1α), PKR-like Endoplasmic Reticulum Kinase (PERK), and Activating Transcription Factor 6 (ATF6). These ER stress sensors possess luminal domains capable of directly or indirectly detecting misfolded proteins when they accumulate to excessive levels [55]. The primary function of these three UPR sensors is to restore the balance between protein folding demand and capacity, enabling the cell to survive and continue functioning. If the amount of misfolded protein is reduced, UPR signaling diminishes, and the cell survives. However, if the adaptive capacity is insufficient to restore protein-folding homeostasis, sustained UPR signaling indicates high or chronic ER stress, potentially leading to cell death [56,57]. An important role in this pathway is also played by Glucose Regulated Protein 78 (GRP78). It is responsible for protein folding and assembly, while preventing the transport of misfolded proteins or protein subunits [58]. GRP78 binds to misfolded and unfolded protein complexes and facilitates their degradation via the ER-associated degradation pathway, which is responsible for regulating the UPR. Under normal cellular homeostasis, GRP78 is bound in its inactive form to ATF6, PERK, and Inositol-Requiring Enzyme 1 (IRE1) [59]. When homeostasis is disrupted, e.g., through nicotine use, GRP78 and PERK eukaryotic translation initiation factor 2α (eIF2α) are increased, along with decreased levels of elastin and collagen I [60].

Activation of the UPR pathway leads to the activation of one and/or all three signaling pathways, which either prevent cell death or amplify it. Upon binding to misfolded proteins, the IRE1α kinase becomes activated and transphosphorylates multiple serine residues on its cytosolic tail, leading to the activation of its adjacent RNase domain. At this point, the downstream consequences depend on the level of ER stress. Under low stress conditions, IRE1α splices an intron from the mRNA encoding the X-box Binding Protein 1 (XBP1) protein, producing the transcription factor XBP1s. This transcription factor causes the expression of many genes that enhance ER size and function, trying to restore cellular homeostasis. However, when stress levels are too high, IRE1α becomes hyperactivated and degrades hundreds of ER-associated mRNAs, depleting ER cargo and exacerbating ER stress. The oligomerization of IRE1α also triggers the activation of apoptosis signal-regulating kinase 1 (ASK1) and its downstream target, c-Jun N-terminal kinase (JNK). Phosphorylation by JNK inhibits the anti-apoptotic protein B-cell leukemia/lymphoma 2 (BCL-2) and initiates a series of reactions aimed at disabling mitochondrial protective proteins like BCL-2. In some cases, this leads to the activation of pro-apoptotic proteins such as BCL-2-associated X (BAX), which permeabilize the outer mitochondrial membrane and release toxic mitochondrial proteins like cytochrome c into the cytoplasm, ultimately leading to cell death [61,62].

Another signaling pathway is associated with the PERK factor, whose activation leads to increased expression of the C/EBP Homologous Protein (CHOP), a gene responsible for growth arrest and DNA damage, as well as a transcription factor that promotes the expression of pro-apoptotic proteins [61,63]. PERK plays a crucial role in mitochondrial function, particularly in maintaining calcium dynamics and regulating metabolism [64]. The absence of PERK leads to ER fragmentation and reduced reactive oxygen species-induced apoptosis, highlighting its role in coordinating communication between the mitochondria and ER and regulating reactive oxygen species-induced apoptosis. PERK activation in this pathway subsequently triggers the activation of ATF4 and consequently CHOP. While this pathway also leads to cell death (as in the IRE1α signaling pathway), the mechanism here involves a different form of cell death—autophagy [65]. CHOP plays a key role in apoptosis by reducing the expression of the anti-apoptotic protein BCL-2, initiating the apoptotic cascade [66].

CHOP activation can occur not only through ATF4 but also via IRE1α, through p38 mitogen-activated protein kinase (MAPK) binding, and in the signaling pathway involving ATF6. After the accumulation of numerous misfolded proteins, ATF6 is transported to the Golgi apparatus, where it is processed by proteases. This process generates the cytosolic fragment of ATF6, which acts as a critical transcription factor, inducing the expression of chaperone genes (proteins responsible for ensuring proper protein folding into the most energetically favorable form), thereby enhancing the protein-folding capacity of the ER [67,68].

Other mechanisms by which the UPR pathway contributes to the pro-apoptotic effects of ER stress involve the three sensors, such as the interruption of protein translation triggered by eIF2α phosphorylation and PERK hyperactivation. This hyperactivation increases the expression of the transcription factor CHOP, which inhibits the expression of the anti-apoptotic gene BCL-2, thereby accelerating cell death [69]. The BCL-2 protein family regulates the intrinsic apoptosis pathway by controlling the integrity of the outer mitochondrial membrane [59,70].

### 3.1. The Role of ER Stress in the Development of Periodontitis—Studies on Cellular Models (Table 2)

Numerous scientific studies on cell models have demonstrated the association of periodontitis with ER stress and the activation of the UPR (Table 2). Specifically, ER stress is linked to the expression of several factors, including CHOP, IRE-1, PERK, ATF-6, and C/EBP β [71]. A study conducted by Bai et al. [70] focused on the latter factor and showed that ER stress induced by the administration of *P. gingivalis* LPS in human periodontal ligament cells (hPDLCs) led to a significantly higher expression of C/EBP β compared to the control group (cells not exposed to LPS). Additionally, activation of the UPR signaling pathway was observed, as indicated by increased levels of PERK, eIF2α, CHOP, and GRP78. The authors also demonstrated that C/EBP β induces the expression of pro-inflammatory cytokines (IL-6 and IL-8) and MMP-8 and MMP-9. These findings suggest that ER stress plays a regulatory role in the inflammatory response and extracellular matrix (ECM) degradation.

**Table 2:** Summary of studies and observed changes in ER stress markers across experimental models. ↑—expression, ↓—inhibition.

Experimental Model	Changes in the Endoplasmic Reticulum Stress Markers	Factors Causing Endoplasmic Reticulum Stress or Other Related	References
Cell Models			
human periodontal ligament cells (hPDLCs)	↑ PERK, ↑ eIF2α, ↑ CHOP, ↑ GRP78, ↑ C/EBP β → ↑ IL-6, ↑ IL-8, ↑ MMP-8, ↑ MMP-9	inflammation through LPS of *P. gingivalis*	Bai et al. [72]
periodontal ligament stem cells (PDLSCs)	↑ CHOP, ↑ ATF4	chemical hypoxia through cobalt chloride (CoCl_2_)	Zheng et al. [73]
human periodontal ligament stem cells (hPDLSCs)	↑ GRP78, ↑ CHOP, ↑ ATF4, ↑ ATF6	inflammation through LPS	Jiang et al. [74]
	↓ GRP78, ↓ CHOP, ↓ ATF4, ↓ ATF6	oridonin	
human periodontal ligament stem cells (PDLSCs)	↑ PERK/eIF2α/ATF4	cyclic tension	Liu et al. [75]
periodontal ligament stem cells (PDLSCs)	↑ PERK, ↑ ATF4, ↑ CHOP	inflammation through TNFα→ ↓ KAT6B	Xue et al. [76]
human periodontal ligament cells (hPDLCs)	↑ PERK, ↑ IRE1, ↑ ATF6 → ↑ CHOP → ↑ caspase-3	inflammation through LPS	Cui et al. [77]
	↓ GRP78 → ↓ caspase-3, ↓ caspase-12	↑ M2 exosomes with melatonin	
human gingival epithelial cells (HGECs)	↑ GRP78, ↑ IRE1, ↑ XBP1	inflammation through LPS	Li et al. [78]

Building on these observations, a study by Zheng et al. [73] highlighted the role of CHOP and activating ATF4 in regulating autophagy-related genes and ER stress-induced inflammation. In this study, periodontal ligament stem cells (PDLSCs) were exposed to cobalt chloride (CoCl_2_), a hypoxia-inducing agent, resulting in significantly increased CHOP and ATF4 expression, along with elevated apoptosis rates compared to the control group of PDLSCs. These results further solidify the connection between ER stress, inflammation, and apoptosis in periodontal cells.

In another study, Jiang et al. [74] investigated the effects of oridonin on inflammation and ER stress in hPDLSCs. Oridonin, a compound with a broad spectrum of pharmacological properties—including anti-inflammatory and hepatoprotective effects—was administered at a concentration of 2 μM, which showed no cytotoxicity for hPDLSCs. The study demonstrated that oridonin significantly mitigated the inhibitory effects of LPS on hPDLSC proliferation and osteogenic differentiation, while promoting both processes by inhibiting the LPS-activated NF-κB/NLRP3 pathway. Furthermore, oridonin alleviated ER stress in LPS-induced hPDLSCs by reducing the expression of GRP78, CHOP, ATF4, and ATF6, suggesting its potential as a therapeutic agent in periodontitis management, as the inhibition of inflammation by oridonin suppressed ER stress.

Similarly, Liu et al. [75] examined the role of the deubiquitinating enzyme USP12 in human periodontal ligament cells (hPDLCs) under mechanical tension and its relationship to ER stress. The bone formation (osteogenesis) of human PDLCs under tension stress is essential for alveolar bone remodeling. The authors in their study showed that cyclic tension leads to ER stress and activates one of the UPR pathways, i.e., the PERK-eIF2α-ATF4 pathway, which increases the expression of osteogenic transcription factors. In turn, inhibition of the deubiquitinating enzyme USP12 increases the accumulation of ubiquitinated proteins and thus induces stronger ER stress, and through the PERK-eIF2α-ATF4 signaling pathway enhances osteogenic differentiation.

A study conducted by Xue et al. [76] utilized factors primarily responsible for periodontitis—IL-1β and TNF-α—both of which have been associated with UPR activation. UPR activation by these cytokines peaked within the first 12 h of exposure and returned to baseline after 24 h, although it did not drop to zero following the removal of the pro-inflammatory factors. According to the authors, this may suggest that prolonged inflammatory stimulation can induce transcriptional changes in periodontal ligament stem cells and activate ER stress. Additionally, the researchers sought to identify critical factors involved in ER stress. They demonstrated that a deficiency of K-lysine acetyltransferase 6B (KAT6B) during chronic inflammation triggers UPR and results in the expression of PERK, ATF4, and CHOP in these cells. These findings confirmed that inflammation reduces the expression of KAT6B, which leads to the activation of UPR. Furthermore, it was shown that chronic inflammation impairs UPR function through KAT6B-dependent PERK transcription, contributing to ER dysfunction, prolonged ER stress, and defective osteogenic differentiation of periodontal ligament stromal cells.

Chronic inflammation increases the expression of GRP78, ATF6, XBP, C/EBP, PERK, and IRE1, as well as caspase-3, which is responsible for apoptosis, and decreases the osteogenic differentiation potential in periodontal tissues. The reduced osteogenic differentiation potential is associated with the weakened ability of cells to differentiate into osteoblasts, which hinders bone regeneration in this disease. A study conducted by Cui et al. [77] demonstrated that activated PERK, IRE1, and ATF6 enhanced expression of CHOP molecules and the transcriptional activity of caspase-3, which mediates the apoptotic cascade by promoting cell apoptosis. In the study, the effect of modified exosomes derived from M2 macrophages on hPDLCs was evaluated. Initially, an inflammatory state was recreated by stimulating the cells with LPS, after which M2 macrophage-derived exosomes were added to the stimulated cells. It was observed that M2 exosomes suppressed ER stress, as evidenced by decreased expression of markers such as GRP78. Additionally, the supplementation of melatonin into these exosomes further reduced the level of ER stress. These findings suggest that M2 macrophage-derived exosomes in combination with melatonin can inhibit the ER stress signaling pathway activated by high LPS concentrations, reverse cell apoptosis (by reducing the expression of caspase-3 and caspase-12 responsible for apoptosis), and improve their impaired osteogenic differentiation.

A study conducted by Li et al. [78] investigated the response of human gingival epithelial cells (HGECs) under high-glucose conditions (5.5 mM and 25 mM glucose) and LPS to simulate the environment of periodontitis in individuals with diabetes. Several key findings emerged from the study. Notably, an increased expression of phosphorylated p65 (p-p65), Nod-like receptor family pyrin domain containing 3 (NLRP3, which triggers immune responses), and IL-1β was observed in cells exposed to high glucose compared to controls, alongside a reduced expression of heat shock protein 47—SERPINH1. The findings demonstrated that SERPINH1 overexpression alleviated prolonged ER stress and inflammation in HGECs under high-glucose conditions. Moreover, in HGECs cultured with high glucose, IRE1α expression and activity were significantly impaired, suggesting defects in this signaling pathway. Treatment with a high concentration of glucose (25 mM) for 48 h resulted in a notable reduction in GRP78 levels compared to cells treated with 5.5 mM glucose. These results highlighted that the IRE1α signaling pathway is inhibited in HGECs under high-glucose conditions, indicating a failure in the ER stress response and UPR. This impairment overwhelms cytoprotective mechanisms, leading to sustained or severe ER stress. Evidence for this includes the downregulation of IRE1α, XBP1-s, and GRP78, showing that UPR functionality is significantly compromised in the presence of hyperglycemia.

### 3.2. The Role of ER Stress in the Development of Periodontitis—Studies on Animal Models (Table 3)

Experimental studies in animal models have also demonstrated a link between UPR activation and development of periodontitis (Table 3). ER stress response is also associated with increased inflammation and bone loss in periodontal tissues, as evidenced by a study conducted by Yamada et al. [79]. In this study, conducted on a group of mice with an induced periodontitis-like condition through oral administration of *P. gingivalis,* the expression of UPR-related molecules and cytokines in gingival tissues, along with the degree of bone loss, were examined. Results obtained through real-time polymerase chain reaction (PCR) analysis of UPR-related gene expression revealed a significant increase in mRNA levels of GRP78 and XBP1. In contrast, the expression of CHOP was not statistically significant. The use of an ER stress inhibitor, such as 4-phenylbutyric acid (4-PBA—a carboxylic acid that, in addition to its ER stress-inhibiting properties, also inhibits cell proliferation, invasion, and migration, while inducing apoptosis, e.g., in glioma cells), suppressed the expression of the aforementioned genes. Histological analysis of bone tissues from the animal models showed increased alveolar bone resorption in mice exposed to *P. gingivalis* compared to the control group, which consisted of mice subjected to sham administration—without oral administration of *P. gingivalis*. This effect was associated with an increased number of osteoclasts in the experimental group. Moreover, significant connective tissue attachment loss, characteristic of periodontitis, was observed in this group, indicated by a substantially greater distance between the cemento-enamel junction (CEJ) and the alveolar bone crest compared to the control group. Another notable difference was the significant increase in IL-6 levels in the experimental group. The administration of 4-PBA attenuated alveolar bone resorption, suggesting that oral administration of *P. gingivalis* enhances UPR activity in gingival tissues. This heightened UPR activity appears to be directly linked to alveolar bone resorption. Supporting this hypothesis, the administration of *P. gingivalis* also increased the expression of several pro-inflammatory cytokines, an effect that was not observed with 4-PBA treatment. Similar studies were conducted by Feng et al. [80], where the inhibitory effect of 4-PBA on the progression of alveolar bone resorption was also demonstrated using a rat model. In this study, rats were treated with LPS to mimic the inflammatory environment.

**Table 3:** Summary of studies and observed changes in ER stress markers across animal models. ↑—expression, ↓—inhibition.

Experimental Model	Changes in the Endoplasmic Reticulum Stress Markers	Factors Causing Endoplasmic Reticulum Stress or Other Related	References
Animal Models			
mice	↑ GRP78, ↑ XBP1	inflammation through oral administration of *P. gingivalis*	Yamada et al. [79]
rats	↑ GRP78, ↑ PERK, ↑ ATF4, ↑ CHOP	LPS	Feng et al. [80]
	↓ GRP78, ↓ PERK, ↓ ATF4, ↓ CHOP	4-PBA + LPS	
rats	↑ CHOP	inflammation through placing 3-O braided silk ligation in the cervical region of the bilateral maxillary second	Tu et al. [81]
mice	↑ ATF6β	inflammation through placing 5-0 silk ligature around teeth	Hayashi et al. [82]

In another study conducted by Tu et al. [81] on rats, it was examined whether cyanidin-3-O-glucoside (C3G), an anthocyanin commonly found in the human diet that exhibits antioxidant properties, could alleviate periodontal damage. The study also aimed to determine whether ER stress plays a role in mitigating periodontitis-related inflammation in rats. Rats were exposed to C3G at varying concentrations—0, 3, and 9 mg/kg—and the expression of three markers—CHOP, phospho c-Jun N-terminal kinase/c-Jun N-terminal kinase (p-JNK/JNK), and NF-κB—was assessed. The expression of all these markers significantly increased in the 0 mg/kg group (group that was not administered with C3G) within the ligation groups (all experimental groups, except the control, were subjected to experimentally induced periodontitis by placing 3-O braided silk ligation in the cervical region of the bilateral maxillary second molars). The administration of C3G resulted in a decrease in the expression of genes associated with ER stress. Additionally, the degree of alveolar bone loss was analyzed in each group using micro-Computed Tomography, which revealed reduced bone loss in the C3G-treated groups (3 mg/kg and 9 mg/kg) compared to the control (group without placing 3-O braided silk ligation and administration of C3G) and 0 mg/kg groups. The findings of this study suggest that periodontitis is induced by ER stress, and factors that alleviate ER stress protect against its development and support the treatment of periodontitis.

Periodontal bone loss and inflammation was induced by a 5-0 silk ligature placed around the teeth, as demonstrated in a study by Hayashi et al. [82]. In this study, the analysis of the ER stress marker ATF6β was conducted in periodontal inflammatory cells from mice and in a periodontal ligament cell line. A significant increase in mRNA expression of ATF6β was observed in the ligatured gingival tissues of mice, particularly in the periodontal ligament and subgingival connective tissue. To reduce ATF6β expression, and thus decrease alveolar bone resorption, the authors used TNF-induced exosomal miR-1260b, a key regulator of periodontal bone tissue loss. Micro-RNA 1260b (miR-1260b) has been shown to reduce ATF6β expression, leading to a decrease in bone resorption. This effect occurs through the regulation of RANKL expression, which is induced by ER stress and controlled by ATF6β. By downregulating ATF6β, miR-1260b suppresses RANKL expression, even in the presence of tunicamycin, a well-known ER stress inducer. The reduction in ATF6β expression also decreased CHOP expression and cleaved caspase-3, while increasing GRP78 expression. Taken together, all these mechanisms also support the hypothesis that reducing the expression of ER stress factors using various substances positively influences the prevention of periodontitis.

### 3.3. The Role of ER Stress in the Development of Periodontitis—Human Studies (Table 4)

ER stress response is also associated with increased inflammation and bone loss in periodontal tissues, as evidenced by a study conducted by Domon et al. on humans (Table 4) [83]. The study aimed to demonstrate the expression of UPR-related molecules in periodontitis and gingivitis. The study included 25 individuals diagnosed with moderate to advanced chronic periodontitis, originally classified according to the 1999 Classification System for Periodontal Diseases and Conditions by the American Academy of Periodontology [84]. Based on the current 2017 Classification of Periodontal and Peri-Implant Diseases and Conditions, these cases would correspond to periodontitis stages 2 to 4, grade A or B [85], from whom tissue samples were collected from both periodontitis and gingivitis sites. Cells were isolated from the tissues and subsequently analyzed. Tissue analysis revealed that the mRNAs for XBP1, ATF4, Selenoprotein S (SEPS1), and CHOP were expressed in both gingivitis and periodontitis tissues. It was also shown that the level of SEPS1 expression was lower than that of XBP1 and ATF4, while CHOP had the lowest expression among the examined genes. The expression levels of XBP1, ATF4, SEPS1, and CHOP were significantly higher in periodontitis lesions compared to gingivitis lesions. The expression of these factors was further compared based on the presence of bacterial LPS from *P. gingivalis* and *Escherichia coli*. For XBP1 mRNA, expression was detected in both cases after 3 h of stimulation, followed by a decline at 24 h. ATF4 and CHOP showed a modest increase in expression at 6 and 12 h in response to *P. gingivalis* LPS, whereas E. coli LPS induced a sustained increase lasting up to 24 h post-stimulation. In contrast, SEPS1 expression showed a slight increase at 6 h, peaking at 12 h before declining by 24 h. This effect was more pronounced with *E. coli* LPS compared to *P. gingivalis* LPS. The study also compared the levels of IL-1β and demonstrated that, at equal concentrations, the stimulatory effect of *E. coli* LPS was stronger than that of *P. gingivalis* LPS. The stimulation with tunicamycin was weaker compared to *P. gingivalis* LPS. However, in all cases, the levels of IL-1β were higher than in the control group. In this study, the expression levels of several UPR-related genes were significantly elevated in the periodontitis group compared to the gingivitis group, suggesting a response driven by ER stress in periodontitis. The upregulation of these transcription factors in periodontitis lesions indicates that periodontal infection may activate an apoptotic pathway. These findings confirm that UPR-related genes exhibit significantly higher expression in disease lesions associated with periodontitis compared to those observed in gingivitis.

**Table 4:** Summary of studies and observed changes in ER stress markers across human models. ↑—expression, ↓—inhibition.

Experimental Model	Changes in the Endoplasmic Reticulum Stress Markers	Factors Causing Endoplasmic Reticulum Stress or Other Related	References
Studies on Human			
Subjects with periodontitis	↑ XBP1, ↑ ATF4, ↑ SEPS1, ↑ CHOP	inflammation through: *E. coli* LPS *P. gingivalis* LPS	Domon et al. [83]

Although the study by Domon et al. (2009) [83] is the only human-based study included in this review, it was selected due to its comprehensive molecular analysis of ER stress markers in periodontal tissues, which remains one of the few studies directly investigating the UPR in human periodontitis and gingivitis. To date, there is a limited number of human studies that explore this specific molecular pathway in the context of periodontal disease. Most of the available data are derived from animal models or in vitro experiments. Despite its publication date, the study by Domon et al. provides uniquely relevant evidence on the clinical expression patterns of UPR-related genes in human periodontal tissues.

## 4. Summary and Conclusions

Inflammatory processes and chemical hypoxia are critical factors contributing to the induction and progression of ER stress, affecting cellular homeostasis and protein folding capacity, as supported by the results of studies that establish a link between ER stress, inflammation, and apoptosis in periodontal cells. Inflammatory stimuli such as LPS, including LPS from *P. gingivalis* (one of the major inducing factors of periodontitis), TNF-α (a factor used to induce inflammation), chemical hypoxia, and placement of 3-0 and 5-0 braided silk ligatures around the neck of the tooth have been shown to induce ER stress in human periodontal ligament cells, periodontal ligament stem cells, and gingival epithelial cells, as well as in mice and rats. In the presented experimental studies, the above-mentioned factors responsible for the development of periodontitis induced ER stress, as observed by the increased expression of UPR pathway proteins such as CHOP, as well as PERK, ATF6 and GRP78.

Interestingly, not all conditions exacerbate ER stress in the same manner. For instance, a hyperglycemic microenvironment was shown to inhibit the IRE1α/XBP1 axis and reduce GRP78 expression, leading to impaired UPR signaling and prolonged ER stress in human gingival epithelium. While this may suggest a distinct mechanism of stress modulation under metabolic conditions, the precise role of hyperglycemia in ER stress regulation remains to be clarified, as its clinical implications are still poorly understood.

To confirm the involvement of ER stress in the pathogenesis of periodontitis, several studies have used ER stress-inhibiting agents—oridonin, melatonin, M2 macrophage-derived exosomes, miR-1260b and 4-PBA. These compounds have been shown to reduce the expression of ER stress markers and consequently prevent the development of periodontitis. Thus, modulating ER stress may offer promising therapeutic potential.

However, ER stress is not universally detrimental. For example, during alveolar bone remodeling, cyclic mechanical stimulation activates the PERK-eIF2α-ATF4 pathway, suggesting that controlled ER stress may play a physiological role in tissue adaptation. Therefore, the inhibition of ER stress should be approached with caution, as it may interfere with beneficial cellular responses depending on the context.

The review is limited by the relatively small number of human studies directly investigating the role of ER stress and UPR pathways in periodontitis. Most of the available data come from in vitro experiments or animal models, which may not fully reflect the complex pathophysiology of human periodontal disease. Additionally, the heterogeneity of study designs, the use of outdated disease classifications in older studies and the limited availability of randomized clinical trials further constrain the ability to draw definitive conclusions. Another limitation is the focus on selected molecular pathways, which, although relevant, may not capture the full spectrum of cellular stress responses involved in periodontal inflammation. Finally, the lack of standardized biomarkers and uniform methodologies across studies poses challenges in synthesizing findings into clear clinical implications.

In conclusion, inflammation emerges as a central mediator in the development of periodontitis, with ER stress acting as a key upstream regulator. While the inhibition of ER stress shows potential as a therapeutic strategy, its dual role—both protective and harmful—necessitates a careful, context-dependent approach. Future research should explore how targeted modulation of ER stress pathways can contribute to effective treatment strategies, ideally combining anti-inflammatory and anti-stress effects without disrupting beneficial physiological processes.

Future in vivo studies in humans are needed to definitively establish the involvement of ER stress in the progression of periodontitis. Furthermore, it is worth considering whether anti-inflammatory therapy alone would be sufficient to reverse the disease-related changes or whether a dual approach targeting both inflammation and ER stress may be necessary. Future studies should lead to the identification of novel therapeutic targets that exert both anti-inflammatory and anti-ER stress effects through distinct pathways, thus contributing to more effective treatment strategies for periodontitis. However, taking all aspects into account, inhibition of ER stress may have serious drawbacks, which requires many studies.

## Figures and Tables

**Table 1:** Summary of systemic risk factors and their impact on periodontitis pathogenesis.

Systemic Factor	Mechanism/Impact on Periodontium	References
Smoking	Enhances pro-inflammatory cytokine production and oxidative stress, thereby promoting periodontal tissue destruction and increasing the risk of tooth loss.	[28,29,30,31,32]
Diabetes mellitus	Alters host immune response and bone metabolism, markedly increasing susceptibility to periodontitis.	[33]
Obesity, cardiovascular disease, dementia	Contribute to disease risk.	[34,35,36]
HIV infection	Predisposes to aggressive forms of periodontitis in younger patients.	[37]

## Abbreviations

The following abbreviations are used in this manuscript. ER—endoplasmic reticulum, UPR—unfolded protein response, CHOP—C/EBP homologous protein, IRE1α—inositol-requiring enzyme 1, PERK—PKR-like endoplasmic reticulum kinase, ATF6—activating transcription factor 6, C/EBP β—CCAAT enhancer-binding protein β, CAL—clinical attachment loss, IgG—immunoglobulin G, TLR—Toll-like receptor, MMP—matrix metalloproteinase, IL—interleukin, LPS—lipopolysaccharide, NADPH—nicotinamide adenine dinucleotide phosphate, HNPs—human neutrophilic α-defensins, HBDs—human β-defensins, IFNγ—interferon-γ, RANK-L—nuclear factor kappa-β ligand, GRP78—glucose regulated protein 78, EIF2α—eukaryotic translation initiation factor 2α, XBP1—X-box binding protein 1, ASK1—apoptosis signal-regulating kinase, JNK—c-Jun N-terminal kinase, BCL-2—B-cell leukemia/lymphoma 2, BAX—BCL-2-associated X, MAPK—mitogen-activated protein kinase, hPDLCs—human periodontal ligament cells, PDLSCs—periodontal ligament stem cells, USP12—ubiquitin-specific protease 12, KAT6B—K-lysine acetyltransferase 6B, HPDLF—human periodontal ligament fibroblast, CD86—cluster of differentiation 86, CCR7—C-C chemokine receptor type 7, dTHP-1—differentiated THP-1, TNF-α—tumor necrosis factor-α, NF-_K_B—nuclear factor kappa-light-chain-enhancer of activated B cells, HGECs—human gingival epithelial cells, p-p65—phosphorylated p65, NLRP3—nod-like receptor family pyrin domain containing 3, PCR—polymerase chain reaction, 4-PBA—4-phenylbutyric acid, CEJ—cemento-enamel junction, C3G—cyanidin-3-O-glucoside, p-JNK/JNK—phospho c-Jun N-terminal kinase/c-Jun N-terminal kinase, MiR-1260b—micro-RNA 1260b, SEPS1—selenoprotein S.
